# Spatio-temporal analysis of mortality among children under the age of five in Manhiça (Mozambique) during the period 1997-2005

**DOI:** 10.1186/1476-072X-10-14

**Published:** 2011-02-18

**Authors:** Geòrgia Escaramís, Josep L Carrasco, John J Aponte, Delino Nhalungo, Ariel Nhacolo, Pedro Alonso, Carlos Ascaso

**Affiliations:** 1 Bioestadística. Departament de Salut Pública. Universitat de Barcelona, Barcelona, Spain; 2 CIBER en Epidemiología y Salud Pública (CIBERESP), Spain; 3 Barcelona Centre for International Health Research (CRESIB), Hospital Clínic/IDIBAPS, Universitat de Barcelona, Spain; 4 Centro de Investigação em Saúde da Manhiça (CISM), Maputo, Mossambique

## Abstract

**Background:**

Reducing childhood mortality is the fourth goal of the Millennium Development Goals agreed at the United Nations Millennium Summit in September 2000. However, childhood mortality in developing countries remains high. Providing an accurate picture of space and time-trend variations in child mortality in a region might generate further ideas for health planning actions to achieve such a reduction. The purpose of this study was to examine the spatio-temporal variation for child mortality rates in Manhiça, a district within the Maputo province of southern rural Mozambique during the period 1997-2005 using a proper generalized linear mixed model.

**Results:**

The results showed that childhood mortality in all the area was modified from year to year describing a convex time-trend but the spatial pattern described by the neighbourhood-specific underlying mortality rates did not change during the entire period from 1997 to 2005, where neighbourhoods with highest risks are situated in the peripheral side of the district. The spatial distribution, though more blurred here, was similar to the spatial distribution of child malaria incidence in the same area. The peak in mortality rates observed in 2001 could have been caused by the precipitation system that started in early February 2000, following which heavy rains flooded parts of Mozambique's southern provinces. However, the mortality rates at the end of the period returned to initial values.

**Conclusions:**

The results of this study suggest that the health intervention programmes established in Manhiça to alleviate the effects of flooding on child mortality should cover a period of around five years and that special attention might be focused on eradicating malaria transmission. These outcomes also suggest the utility of suitably modelling space-time trend variations in a region when a point effect of an environmental factor affects all the study area.

## Background

Reducing childhood mortality (mortality before the age of five) is the fourth goal of the Millennium Development Goals agreed at the United Nations Millennium Summit in September 2000. However, childhood mortality in developing countries remains high: it has been estimated that about 10.6 million children die each year, although most of these deaths could be prevented by public health interventions [1-3]. Therefore, the quality of a health system in a country or region is a crucial issue when it comes to ensuring the success of health campaigns developed to decrease such mortality. Unfortunately, most developing countries have poorly-functioning systems for monitoring life events; for example, in Africa only 30% of births are registered [4].

Manhiça, a district within the Maputo province of southern rural Mozambique, is covered by a continuous demographic surveillance system (DSS) that since 1996 has been run by the Health Research Centre of Manhiça (CISM). Details about the census and follow-up procedures are described by Alonso *et al. *[[5], p. 189-195]. The DSS has proved to be an adequate tool for reporting accurate demographic measures for southern rural Mozambique [6]. Moreover, providing an accurate picture of space-time variations in child mortality in the district might generate further ideas for health planning actions in Manhiça. In this regard, modelling the temporal variability in childhood mortality would help health researchers to understand the relationship between this mortality and events that may vary from year to year, for example, climatic conditions. In terms of spatial variability, areas with high mortality risks would require special health strategies that are different to those in areas with lower risks.

In spatial mortality mapping the data used are often aggregated and comprise death counts within a lattice, which might be health areas or administrative zones. It should be noted here that when the spatial lattice involves small areas the use of crude measures such as the standardised mortality rate is likely to yield unacceptably large standard errors or suggest extreme risks that misrepresent the real risk heterogeneity among areas and time [7]. These problems are usually overcome by applying smoothing methods such as the Poisson regression mixed model (PRMM) [8]. The PRMM includes a random effect for each area under study with a certain prior distribution that can accommodate the real risk heterogeneity among areas, or the spatial dependence of the data due to common unmeasured risk factors which contiguous areas might share. A widespread prior choice for the random effects in this kind of study is what is known as the conditional autoregressive model [9,10] (PRMM-CAR). If in addition of spatial variability one aims to evaluate the time-trend evolution of these areas one can extend the PRMM-CAR model by including a plausible function depending on time in the linear predictor of the model. Bernardinelli *et al. *[7] extended the PRMM-CAR model to consider a linear time trend in their analysis. The authors proposed to include random effects for both the area-specific intercept and time trends. By including a linear time trend in space the estimates of an area-specific trend can borrow strength from related areas, thus resulting in more sensitive estimates when data are scarce.

The present study evaluates the spatio-temporal variation in childhood mortality in the village of Manhiça and its outskirts during the period 1997-2005, using a modified version of the space-time trend variation model proposed by Bernardinelli *et al. *[7] to model appropriately our mortality data.

## Methods

### Study area

The study was conducted in the village of Manhiça and its outer reaches which is located in Maputo province, southern Mozambique, during the period January 1997 to December 2005.

The village of Manhiça, 25°24'S and 32°48'E, is situated in an area of flat, bush savannah along the coast of Mozambique; it is 80 km north of the capital Maputo and covers approximately 120 km^2^. Geographic characteristics, climatological conditions and population living customs in Manhiça have been described in detail by Abellana *et al. *[11].

The study area is subdivided for political-administrative reasons into 115 small neighbourhoods. Maps of the area were obtained from the Direção Nacional de Geografia e Cadastro of Mozambique (reference numbers: SG-36/I-I-NE page 1169 2532, SG-36/J-I-NO page 1170 2533A1, SE-26/V-III-SO page 1178 2532B3, SG-36/I-IV-SO page 1179 2532B4). The maps were scanned and the limits of the neighbourhoods were georeferenced by the Manhiça Health Research Centre (CISM).

### Study subjects

Data were collected via the demographic surveillance system (DSS) which has been operating and run by the CISM in Manhiça since 1996. During surveillance every household is visited at least twice a year and life events including births, deaths and migrations are recorded. Other events such as pregnancies, abortions, stillbirths and level of education are also documented during the visits, as are the characteristics of household construction. Since six-monthly visits could lead to the omission of some events the information recorded was complemented through weekly updates by the key informants in the community, as well as via daily hospital visits.

The information collected from the DSS regarding children consisted in birth date, gender, neighbourhood of residence, and, in the case of death, the date of this event.

Each child included in the analysis had to have resided in the study area during the period 1997-2005, while the time at risk for each child in each year was defined as the number of days that the child was alive during that year and aged below five years old.

We also collected socio-demographic indicators at the neighbourhood level, for example, the percentage of illiterate mothers in the specific neighbourhood, percentage of households with a WC, percentage of households with a kitchen, percentage of cane households and percentage of households with a single construction. These covariates were categorised as quartiles to facilitate the identification of any association patterns with the mortality rates.

### Standardised mortality rates

Annual standardised mortality ratios (SMR) at both district (i.e., Manhiça and its outskirts) and neighbourhood level were determined. To obtain the annual expected number of deaths, gender-specific mortality rates (calculated as the number of deaths divided by the number of children-per-day at risk in each gender group in the specific year under consideration when district level is the goal, or year and neighbourhood when neighbourhood level is the objective) were multiplied by the sum of children-per-day at risk in each gender group across the whole period from 1997 to 2005 and across all neighbourhoods. Therefore, SMRs that deviate from 1 show which years, or neighbourhoods in a specific year, have higher/lower mortality rates than the constant average across all the study area and over the period analysed.

### Exploratory analysis

#### Global time-trend

For modelling purposes one usually assumes that death counts are Poisson distributed. Therefore, generalised linear models (GLM) are applied, where the canonical function that links death counts to the linear predictor is the logarithm, and the log-expected counts are included into the model as an offset. This model is commonly termed the Poisson regression model (PRM). Hence, we conducted a time-trend exploratory analysis by plotting the log-transformed SMRs (log*SMR*) for each year for the district of Manhiça revealing a marked increase followed by decreased shape of the crude log*SMR*, thus indicating that the most plausible model includes time in the analysis by using a second-order polynomial time trend, i.e. log *SMR *= *f*(*t*,**α**) = *α*_0 _+ *α*_1_*t *+ *α*_2_*t*^2^.

Therefore, if *Y*_*it *_and *E*_*it *_are the number of deaths and expected counts respectively, in the *i*-th neighbourhood and *t*-th year, and we denote by *μ*_*it *_the expected value of *Y*_*it *_, i.e., *μ*_*it *_= *E*(*Y*_*it*_), then the PRM (which will be referred as Model 0) is defined as:

$$\log\left(\mu_{it}\right)=\log\left(E_{it}\right)+\alpha_{0}+\alpha_{1}t+\alpha_{2}t^{2}+{\text{x}}_{i}\beta$$

where *α*_0 _represents the baseline log-relative mortality risk across all neighbourhoods and *α*_1 _and *α*_2 _stand for the global log-relative mortality rate evolution. In order to avoid possible colinearity between *α*_1 _and *α*_2 _in model parameter estimates, *t *was constructed to range discretely from -4 to 4, and thus *t *and *t*^2 ^are orthogonal vectors. This is simply a year translation in which the mid-point of the period, 2001, is taken as the translation value. Thus, 2001 becomes the artificial value of 0 and therefore the baseline year in the analysis, while -4 corresponds to 1997 and 4 to 2005. Note that if *α*_1 _and *α*_2 _are estimated to be 0 it would mean that no changes in mortality rates would be observed across the period. If only *α*_2 _is estimated to be 0 a linear time-trend would be observed, and the sign of *α*_1 _would indicate whether there had been a linear increase or decrease in mortality rates. Otherwise, if *α*_2 _is estimated to be significantly different from 0 then a curved evolution is detected, and its sign stands for the convexity or concavity of the time evolution in mortality rates.

Finally, **β **is the vector of covariate parameters (in our analysis correspond to the socio-demographic indicators) and **x**_i _corresponds to the *i*-th row of the design matrix. The exponential of **β **(e^**β**^) is referred as the covariate-specific relative risk. Likelihood-ratio tests were used to decide which socio-demographic indicators would be included in the final model.

#### Spatio-temporal variability

In order to explore whether there was a common time-trend function or whether there were some neighbourhoods in which specific time-trends deviated from this global tendency, a PRM including the time-trend function for each neighbourhood was conducted. Here, therefore, the dependent variable was the number of deaths per year in the specific neighbourhood under consideration.

From the 115 PRM models adjusted separately for each neighbourhood we obtained 115 estimates about intercept (*α*_0_), first- (*α*_1_) and second-order (*α*_2_) polynomial coefficient estimates, thus describing the specific time-trend for each neighbourhood. When a high variability was observed this indicated the need to include random effects in the PRM.

To explore the possible spatial dependency among neighbourhoods for both neighbourhood-specific intercepts and neighbourhood-specific time trend we calculated the Moran I test [12] for spatial autocorrelation among the 115 estimates of each element of the **α **vector (*α*_0_, *α*_1 _and *α*_2 _respectively). If this spatial dependence assumption was not reasonable, random effects were given an exchangeable prior function, i.e. random noise; otherwise, when a significant result was observed this recommended the inclusion of spatially-dependent random effects.

### Data modelling: Poisson regression models with random effects

Following the exploratory results we applied Poisson regression mixed models (PRMM) accounting for neighbourhood-specific random effects.

If we assume that the conditional mean mortality count for the *i*-th neighbourhood and *t*-th year given the random effect vectors **b **= (**b**_1_,...,**b**_*J*_), with *J *being the number of random effects vectors to be considered in the analysis, is given by *μ*_*it *_= *E*(*Y*_*it *_| **b**), then the PRMM is specified as:

$$\log\left(\mu_{it}\right)=\log\left(E_{it}\right)+f\left(t,\alpha ,\text{b}\right)+{\text{x}}_{\text{i}}\beta$$

In the event of spatial dependence we applied the improper conditional autoregressive joint distribution (ICAR) [13,14] which induces 'local' smoothing by complete borrowing strength from related neighbours:

$${\text{b}}_{j}=\left(b_{j,1},\cdots ,b_{j,115}\right)\text{\textasciitilde{}MVN}\left(0,\sigma_{S_{j}}^{2}{\text{Q}}^{-}\right)$$

where *Q *is the 115 × 115 matrix defining the neighbourhood structure. Here we adopted the common neighbourhood approach, which defines areas *i *and *k *as neighbours if they share a common boundary, meaning that a specific neighbourhood will borrow strength from contiguous neighbourhoods. Therefore, the diagonal elements of *Q *are equal to the number of neighbours of the specific neighbourhood, while off-diagonal elements will be -1 if the corresponding areas are neighbours and 0 otherwise. $\sigma_{S_{j}}^{2}$ is the variance component reflecting the spatially-structured variability.

In the event that spatial dependency was not reasonable exchangeable priors were applied:

$${\text{b}}_{j}=\left(b_{j,1},\cdots ,b_{j,115}\right)\text{\textasciitilde{}MVN}\left(0,\sigma_{H_{j}}^{2}\text{I}\right)$$

where **I **is the 115 × 115 identity matrix and $\sigma_{H_{j}}^{2}$ a non-structured variance component.

Three different PRMMs accounting for a second order polynomial time-trend and neighbourhood-specific random effects were applied. The first PRMM (Model 1) included random effects for neighbourhood-specific intercepts with non-structured variability. The second PRMM (Model 2) accounted for neighbourhood-specific intercept random effects with ICAR prior function. Model 1 and 2 are defined as follows, and the difference relies in the joint distributional assumption of the random effects, exchangeable and ICAR for Model 1 and 2 respectively:

$$\log\left(\mu_{it}\right)=\log\left(E_{it}\right)+\alpha_{0}+b_{0i}+\alpha_{1}t+\alpha_{2}t^{2}+{\text{x}}_{i}\beta$$

Here, *b*_0i _represents the underlying log-relative mortality risk for the *i*-th neighbourhood in the baseline year 2001, however, since no other random effects are included into the model and *b*_0i _are independent from both time-trend and covariates, *b*_0i _are interpreted as the underlying log-relative mortality risks constant for the whole period considered.

A third model (Model 3) was also conducted which accounted as well for curvature time-trend random effects with exchangeable prior functions:

$$\log\left(\mu_{it}\right)=\log\left(E_{it}\right)+\alpha_{0}+b_{0i}+\left(\alpha_{1}+b_{1i}\right)t+\left(\alpha_{2}+b_{2i}\right)t^{2}+{\text{x}}_{i}\beta$$

were *b*_1i _and *b*_2i _are the neighbourhood-specific deviation trends from the overall trend explained by the fixed effects *α*_1 _and *α*_2_. Note that predictions about *α*_2 _+ *b*_2__*i *_illustrate the sharpness of the neighbourhood-specific curvatures.

The exponential of the sum of all elements in the linear predictor of a PRMM is referred as the neighbourhood-specific relative risk in the explicit year.

The PRMM parameters were estimated using the *penalised quasi-likelihood *(PQL) approach [15].

For inference about neighbourhood-specific underlying relative risks, prediction intervals about the random effects predictions were applied according to the approach proposed by Escaramís *et al. *[16]. When predictions of the random effects are significantly different from 0, the prediction of the underlying neighbourhood-specific relative risk will have a 100(1-0.05)% prediction interval entirely above or below 1, where 0.05 is the type I error.

The quasi-likelihood version of the Schwarz (BIC) information criterion [17] was used as a rule of thumb to discriminate between models [[18] (sect. 6.4), 19 (sect. 15.2)]. We also estimated the dispersion parameter of the resulting PRMM. This parameter estimate is a goodness-of-fit measure in Poisson regression models, since it tells us how close the theoretical variability of the variable analysed is to the estimated variability through the model. Therefore, if both variabilities are equal then the dispersion parameter should be estimated to be 1, and therefore the distributional assumption of the model holds.

Model 3 is a modified version of the space-time trend variation model proposed by Bernardinelli *et al. *[7] in the sense that incorporates a convex time-trend via a second-order polynomial specification in the linear predictor instead of a linear time-trend, thus letting the model appropriately fit the steep increase followed by the steep decrease of the global time-trend evolution of our mortality data. Furthermore Bernardinelli *et al. *[7] argue the use of full Bayesian approaches, such as Markov Chain Monte Carlo (MCMC), for model parameter estimates in order to take into account the uncertainty in inference about underlying relative-risks predictions that arises from having to estimate variance components instead of plugging-in true values into the predictors. We have here taken benefit of the PQL technique in terms of lower CPU time consumption against MCMC methods by using Escaramís *et al*. [16] approach for inference about underlying relative-risk predictions. This approach is based on an analytical adjustment of such uncertainty and has been shown to provide accurate interval predictions when using the PQL technique to obtain model parameter estimates.

The models were fitted using the SAS software with the GLIMMIX procedure [20] and details on the syntax used are given in Additional file 1.

## Results

### Data description

The mean population count for the study area over the period considered was 9013 children below the age of five (range: 7287 in 1997 to 10,025 in 2004). Population per neighbourhood ranged from a mean of 18 children per year to a mean of 499.

The mortality rate reached a peak of 42 cases per 1000 children/year in 2001, with the lowest values being found at either end of the period (27 and 26 cases per 1000 children/year in 1997 and 2005, respectively).

Of all the socio-demographic indicators considered in our study only *households with a single construction *showed a significant association: those neighbourhoods with a lower percentage of single constructions showed an increased effect on child mortality rates (Table 1).

**Table 1:** Associations between log standardised mortality rates (log of observed cases/expected cases) for children and socio-demographic indicators adjusted by global mean time-trend among the 115 Manhiça neighbourhoods derived from the Poisson regression models.

Covariates	Categories	Estimate	p-value
		(standard error)	
Time	Continuous	0.005 (0.009)	0.5736
time^2^		-0.027 (0.004)	<0.0001

% households with WC	<74%	0.170 (0.095)	0.3546
	74-82%	0.080 (0.077)	
	82-89%	0.075 (0.067)	
	>89%		

% households with kitchen	<40%	-0.094 (0.099)	0.5196
	40-49%	-0.086 (0.080)	
	49-59%	-0.001 (0.069)	
	>59%		

% cane households	<62%	-0.065 (0.086)	0.1963
	62-72%	0.090 (0.067)	
	72-81%	0.008 (0.060)	
	>81%		

% households with a single construction	<54%	0.213 (0.072)	0.0003
	54-60%	0.023 (0.070)	
	60-67%	-0.080 (0.066)	
	>67%		

% households with an illiterate mother	<49%	0.005 (0.090)	0.4626
	49-54%	0.009 (0.082)	
	54-59%	0.088 (0.071)	
	>59%		

**Figure 1:**
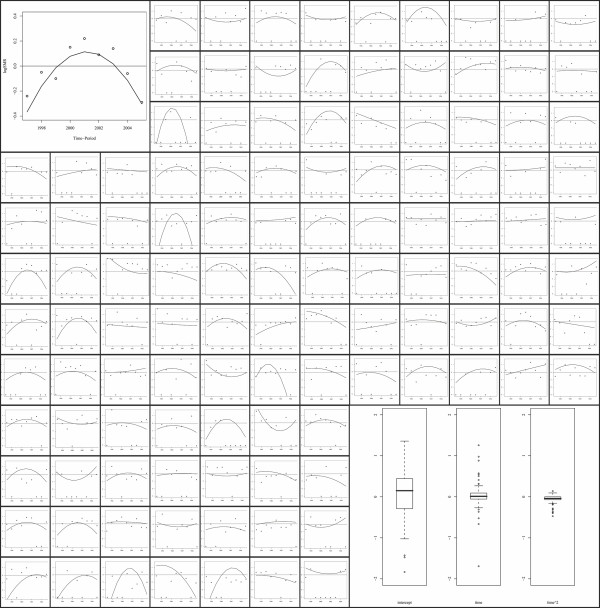
**First plot: dots indicate annual log standardised mortality rates for Manhiça district, while the smoothed line is the estimated second-order polynomial time trend derived from the Poisson regression model**. The same information is shown in subsequent plots per each of the 115 neighbourhoods that comprise Manhiça. Last plot: boxplots showing the variability among the 115 intercept, first- and second-order polynomial estimates derived from the 115 neighbourhood-specific Poisson regression models.

Exploratory results for our data showed a mean time-trend convex curvature among southern Manhiça neighbourhoods associated with the crude *log*SMR and revealing a marked increase followed by decreased shape (Figure 1, first plot in the grid). The estimated second-order polynomial time-trend confirmed this result as the sign of the quadratic term is negative (Table 1 and Figure 1).

Time evolutions of the crude *log*SMRs for each of the 115 neighbourhoods are also shown in Figure 1. The plots include as well the smoothed time-trends derived from fitting the PRM for each neighbourhood. A high variability between trends is perceptible, however a high proportion of them seem to have undergone a convex time evolution in mortality rates. Boxplots in the last cell of the grid of Figure 1 are a resume of these neighbourhood-specific smoothed time-trends; in particular the plots show the variability between neighbourhoods through their intercept, first- and second-order polynomial estimates. A high level of heterogeneity across neighbourhoods can be observed for the intercept estimates; however a lower variability is shown for the first- and second-order estimates. These results indicate the need to include random effects in the neighbourhood-specific intercept in the final model but time trend appears to be constant across all neighbourhoods.

Furthermore, the 115 intercept estimates showed a significant autocorrelation (Moran's I = 4.1, p < 0.0001), whereas the 115 first- and second-order polynomial estimates did not (Moran's I = 1.181 and 0.224, respectively, with p = 0.119 and 0.411, respectively). These results suggested the presence of a common spatial pattern in the whole period analysed as the geographic variability of the intercept represents the baseline log-mortality rate variability which is time independent. However the non-significant autocorrelation for the first- and second-order estimates indicated that the time-trend pattern of a specific neighbourhood may not depend on time-trend patterns from related neighbourhoods.

### Data modelling

Results from the three PRMM models as well as the model without random effects (Model 0) are shown in Table 2. Model 0 exhibits over-dispersion (ϕ = 1.218) which is clearly addressed by extending the PRM by including random effects in the linear predictor (Models 1, 2 and 3). According to the *quasi-*BIC, Model 2 seems to capture the variability structure of the data better than Model 1 (BIC for Model 1 = 2713.22 vs BIC for Model 2 = 2704.94) revealing therefore that a spatial structure among neighbourhoods is present in childhood mortality rates in southern Manhiça. Model 3 extends Model 2 to take into account the time-trend variability in mortality rates among neighbourhoods. According to the results from this last model, the variability of the time-trend curvature among neighbourhoods is negligible since the variance for the first- and second-order polynomial random effects is almost 0. In addition the *quasi*BIC of Model 3 (BIC = 2711.24) is greater than the *quasi*BIC of Model 2, suggesting the use of Model 2 to analyse the data under the parsimonious rule.

**Table 2:** Model parameter estimates derived from the Poisson regression mixed models used to evaluate the spatio-temporal variation in Manhiça child mortality rates (log standardised mortality rates; log of observed cases/expected cases) during the period 1997-2005.

Parameter symbol	Description of parameter		Estimate			
			(Standard Error)			
			
			Model 0*	Model 1	Model2	Model 3
α_0_	Baseline log-rate across all areas		0.101 (0.051)	0.129 (0.063)	0.114 (0.062)	0.114 (0.062)

α_1_	Log of the overall curvature trend during the period 1997-2005		0.009 (0.010)	0.008 (0.010)	0.008 (0.010)	0.008 (0.010)
α_2_			-0.028 (0.004)	-0.028 (0.004)	-0.028 (0.004)	-0.028 (0.004)

**β**	Log-relative risks of % households with a single construction	<54%	0.276 (0.068)	0.231 (0.085)	0.224 (0.084)	0.220 (0.084)
		54-60%	0.090 (0.069)	0.052 (0.082)	0.050 (0.082)	0.051 (0.082)
		60-67%	-0.031 (0.067)	-0.060 (0.076)	-0.059 (0.076)	-0.056 (0.076)
		>67%	baseline	baseline	baseline	baseline

$\sigma_{H}^{2}$	Variance component reflecting the non structured variability of neighbourhood-specific intercepts		--	0.051 (0.017)	--	--

$\sigma_{S}^{2}$	Variance component reflecting the spatially-structured variability of neighbourhood-specific intercepts		--	--	0.094 (0.030)	0.092 (0.032)

$\sigma_{time}^{2}$	Variance components reflecting the non-structured variability of neighbourhood-specific curvature time trend		--	--	--	0.0006 (0.0013)
$\sigma_{time^{2}}^{2}$			--	--	--	0.00008 (0.0002)

ϕ	Dispersion parameter		1.218	1.067	1.057	1.036

*quasi*BIC	*Quasi-likelihood *Information criteria		3394.66*	2713.22	2704.94	2711.24

*Model 0 is a Poisson regression model, which does not include random effects, the coefficient estimates are maximum likelihood estimates and the goodness of fit measure here is the Schwarz information criterion (BIC) based on the likelihood instead of the quasi-likelihood.

Therefore Model 2 is selected to describe the data. This model shows to appropriately fit the theoretical Poisson variability of mortality counts as the dispersion parameter is very close to 1 (ϕ = 1.057). According to the model those neighbourhoods with the lowest percentage of households with a single construction had a significant risk effect on child mortality rates. The overall trend in child mortality rates for the period studied took a convex form, as the second-order polynomial time-trend estimate is negative (α_2 _= -0.028(0.004)). The model also shows that the same spatially-structured variability among neighbourhoods is present in the whole period analysed ($\sigma_{S}^{2}$ = 0.094(0.030)). Therefore neighbourhoods that started with lower mortality rates in 1997 reached lower mortality rates in the middle of the period (2001) and reverse.

**Figure 2:**
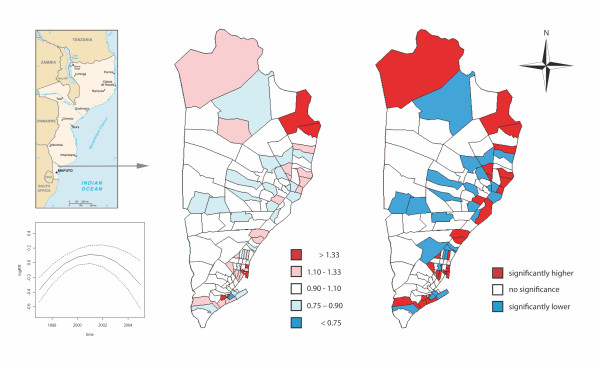
**Left-bottom panel: the estimated second-order polynomial time trend derived from the Poisson regression mixed model; dashed lines correspond to the 95% confidence intervals of the estimated time trend curvature**. Left-hand side map represents point predictions of underlying relative risks for child mortality in Manhiça district common in the entire period from 1997 to 2005, derived from the Poisson regression mixed model. Right-hand side map shows the significance of the underlying relative risks with a 90% of confidence.

Figure 2 shows the two independent components of the model, the convex time-trend common for all neighbourhoods (bottom-left panel) and the common spatial pattern along the whole period (maps in right-hand side), where neighbourhoods with highest underlying relative risks are situated in the peripheral side of the district.

**Figure 3:**
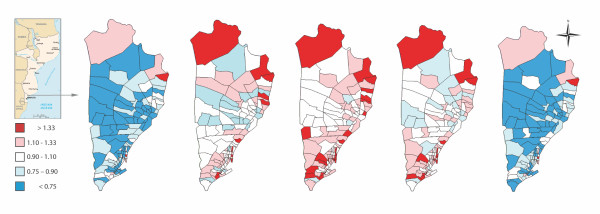
**Point predictions of relative risks for child mortality in Manhiça district, derived from the Poisson regression mixed model corresponding from left to right to: 1997, 1999, 2001, 2003 and 2005**.

Figure 3 describes the spatio-temporal variability of the neighbourhood-specific relative risks. For the sake of simplicity maps for the years 1997, 1999, 2001, 2003 and 2005 are shown, while the remaining years in the study period are taken as intermediate steps. It can be seen that the highest rates of child mortality are reached in 2001, although the situation returns to initial values in 2005, with a very similar spatial pattern along the whole period analysed, where the biggest cluster with lower relative risks is situated in the west-central side of the district. The slight difference of the spatial pattern of the relative risks across years is due to the little changes of the socio-demographic indicators during the period under consideration.

## Discussion

The present study evaluated the space-time trend variations in the southern area of Manhiça district for the period 1997-2005.

The data were analysed using a Poisson regression mixed model (PRMM) that included a second-order polynomial time trend. This model showed a correct fit to the steep increase from 1997 to 2001 followed by a steep decrease thereafter of the global mortality rates among the 115 neighbourhoods that comprise the southern Manhiça district. This model is similar to the linear space-time trend variation model proposed by Bernardinelli *et al. *[7], which was also applied by Kleinsmidth *et al. *[21] to evaluate trends in malaria incidence rates in a small area in South Africa. Our model includes random effects for neighbourhood-specific intercepts accounting for spatial dependence; however we did not find neighbourhood-specific variability on time-trends. The inclusion of the spatial random effects with respect to baseline enables the data for each area to borrow strength from related areas across the whole period. The model can also readily detect those neighbourhoods with extreme underlying mortality risks present in all the study period by constructing prediction intervals of the neighbourhood-specific random effect predictions. The underlying relative risks reflect the mortality risks that are not explained by known risk factors and the fact that they present a spatial structure also reflect unknown common environmental factors that contiguous neighbourhoods share.

Our model shows that a spatial distribution of childhood mortality is patent in all the study period, as shown in Figures 2 and 3. The neighbourhoods with the lowest rates are mainly clustered in the centre-west side of the district, and the tendency is towards higher in the periphery areas. This is better seen in Figure 2 map on the left-hand side where significantly higher underlying relative risks are all situated in the peripheral side of the district. Despite being more blurred in our mortality data, this spatial pattern is similar to the marked spatial pattern described by Abellana *et al. *[11] for the spatial distribution of malaria infection in children under the age of 10 years living in the area. These authors also found that children under five years of age are at the highest risk group of malaria infection. This is in accordance with the fact that a high percent of deaths of sub-Saharan children under the age of five is attributable to malaria [22]. Concretely, malaria due to *Plasmodium falciparum *is the main killer among children between 28 days to 4 years living in southern Manhiça [23]. However childhood deaths in the study area are also attributable to other causes such as malnutrition, diarrhoea, pneumonia or HIV/AIDS [23] that might spoil the specific spatial structure of malaria infection in children living in southern Manhiça.

**Figure 4:**
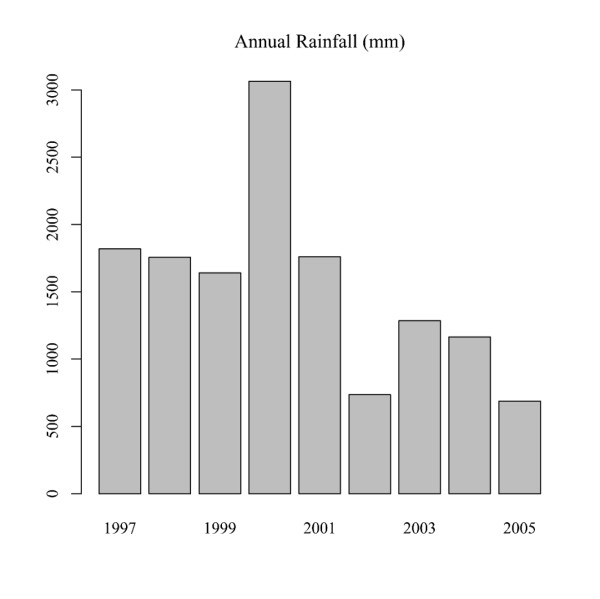
Annual rainfall (mm) in Manhiça district during the period 1997-2005

The model also shows that all southern Manhiça has undergone a convex evolution in child mortality rates during the period 1997-2005, with a maximum being reached in 2001. This convexity pattern could have been caused by the precipitation system that started in early February 2000, and which saw heavy rain flood parts of Mozambique's southern provinces. In as little as three weeks the main river systems in Mozambique, from the Incomati River in the south to the Zambézia River in the centre-north, exceeded their normal yearly flood rates by several fold. As a result, the surrounding villages were flooded, causing the worst damage in fifty years. Manhiça is crossed by the Incomati River from north to south and was one of the areas severely affected by the precipitation system. As can be seen in Figure 4 the annual rainfall in 2000 was markedly higher than the other annual rainfalls for the period analysed, reaching values above 3000 mm, whereas in other years it did not even reach 2000 mm. Rainfall was recorded by the climatology station located near CISM. Given that many of the causes of global child mortality in developing countries, including malaria, diarrhoea, acute respiratory infections and malnutrition, are highly sensitive to climatic conditions such as flooding, this may explain the higher mortality rates in the middle of our study period.

## Conclusions

The study showed that the same geographical pattern of underlying childhood mortality rates was patent in Manhiça and its outskirts in all the study period from 1997 to 2005 and similar to the spatial distribution of childhood malaria infection, suggesting that special attention might be focused on eradicating malaria transmission in Manhiça to achieve the reduction of childhood mortality agreed in the Millennium Development Goals at the United Nations Millennium Summit in September 2000 [24].

The study also showed that childhood mortality in Manhiça was modified from year to year describing a convex time-trend during the period, a common pattern for all the neighbourhoods that comprise the area, where the peak coincides with the precipitation system that started in early February 2000. However after approximately five years child mortality rates returned to initial values suggesting that health intervention programmes established in Manhiça to alleviate the effects of flooding on child mortality should cover a period of around five years. These outcomes suggest as well the utility of modelling space-time trend variations in a region when a point effect of an environmental factor affects all the study area.

## Abbreviations

CAR: conditional autoregressive model; CISM: Manhiça Health Research Centre; PRMM: Poisson regression mixed model; SMR: standardize mortality ratio;

## Competing interests

The authors declare that they have no competing interests.

## Authors' contributions

GE, JLC and CA were responsible for statistical analysis and the preparation of the manuscript. JJA and PA conceived and designed the study. DN and AN were responsible for fieldwork and the geographical information system of Manhiça. JJA was responsible for data cleaning and manuscript preparation.

All authors read and approved the final manuscript.

## Supplementary Material

Additional file 1 **SAS syntax details**. SAS syntax details for the Poisson regression mixed models parameter estimates. Click here for file

## References

[B1] Victora CG Wagstaff A Schellenberg JA Gwatkin D Claeson M Habicht JP Applying an equity lens to child health and mortality: more of the same is not enough Lancet 2003 362 233 241 10.1016/S0140-6736(03)13917-7 12885488

[B2] Bryce J Boschi-Pinto C Shibuya K Black RE WHO estimates of the causes of death in children Lancet 2005 365 1147 1152 10.1016/S0140-6736(05)71877-8 15794969

[B3] Jones G Steketee RW Black RE Bhutta ZA Morris SS How many child deaths can we prevent this year? Lancet 2003 362 65 71 10.1016/S0140-6736(03)13811-1 12853204

[B4] Stansfeld SK Walsh J Prate N Evans T Information to improve decision making for health. In Disease control priorities in developing countries 2006 New York, Oxford University Press 1017 30

[B5] Alonso P Saute F Aponte JJ Population and health in developing countries; vol.1, population, health and survival at INDEPTH sites 2002 Ottawa: International Development Research Centre (IDCR)

[B6] Nhacolo AQ Nhalungo DA Sacoor CN Aponte JJ Thompson R Alonso P Levels and trends of demographic indices in southern rural Mozambique: evidence from demographic surveillance in Manhiça district BMC Public Health 2006 6 291 10.1186/1471-2458-6-291 17137494 PMC1712340

[B7] Bernadinelli L Clayton D Pascutto C Montomoli C Ghislandi M Songini M Bayesian analysis of space-time variation in disease risk Statistics in Medicine 1995 14 2433 43 10.1002/sim.4780142112 8711279

[B8] McCulloch CE Searle SR Generalized, Linear, and Mixed Models 2001 Canada: Wiley Series in Probability and Statistics

[B9] Besag J York J Mollié A Bayesian image restoration, with applications in spatial statistics Ann I Stat Math 1991 43 1 59 10.1007/BF00116466

[B10] Pascutto C Wakfield JC Best NG Richardson S Bernardinelli L Staines A Elliott P Statistical issues in the analysis of disease mapping data Statistics in Medicine 2000 19 2493 2519 10.1002/1097-0258(20000915/30)19:17/18<2493::AID-SIM584>3.0.CO;2-D 10960868

[B11] Abellana R Ascaso C Aponte J Saute F Nhalungo D Nhacolo A Alonso P Spatio-seasonal modelling of the incidence rate of Malaria in Mozambique Malaria Journal 2008 7 228 10.1186/1475-2875-7-228 18976458 PMC2584655

[B12] Moran PAP A test for spatial independence of residuals Biometrika 1950 37 178 181 15420264

[B13] Besag J Spatial interaction and the statistical analysis of lattice systems J R Stat Soc B 1974 36 192 236

[B14] Sun D Tsutakawa RK Speckman PL Posterior distribution of Hierarchical Models using CAR(1) Distributions Biometrica 1999 86 341 350 10.1093/biomet/86.2.341

[B15] Breslow NE Clayton DG Approximate inference in generalized linear mixed models Journal of the American Statistical Association 1993 88 9 25 10.2307/2290687

[B16] Escaramis G Carrasco JL Ascaso C Detection of Significant Disease Risks Using a Spatial Conditional Autoregressive Model Biometrics 2008 64 1043 1053 10.1111/j.1541-0420.2007.00981.x 18261162

[B17] Schwarz MD Estimating the Dimension of a Model Annals of Statistics 1978 6 461 464 10.1214/aos/1176344136

[B18] Verbeke G Molenberghs G Linear Mixed Models for Longitudinal Data 2000 New York: Springer-Verlag

[B19] Molenberghs G Verbeke G Models for Discrete Longitudinal Data 2005 New York: Springer

[B20] Littell RC Milliken GA Stroup WW SAS® for Mixed Models 2006 2 Cary, NC: SAS Institute Inc

[B21] Kleinschmidt I Sahrp B Mueller I Vounatsou P Rise in Malaria Incidence Rates in South Africa: A small-Area Spatial Analysis of Variation in Time Trends American Journal of Epidemiology 2002 155 3 257 264 10.1093/aje/155.3.257 11821251

[B22] Rowe AK Rowe SY Snow RW Korenromp EL Schellenberg JRMA Stein C Nahlen B Bryce J Black RE Steketee RW The burden of Malaria mortality among African children in the year 2000 International Journal of Epidemiology 2006 35 3 691 704 10.1093/ije/dyl027 16507643 PMC3672849

[B23] Sacarlal J Nhacolo AQ Sigauque B Nhalungo DA Abacassamo F Sacoor CN Aide P Machevo S Nhampossa T Macete EV Bassat Q David C Bardaji A Letang E Saute F Aponte JJ Thompson R Alonso PL A 10 year study of the cause of death in children under 15 years in Manhiça, Mozambique BMC Public Health 2009 9 67 10.1186/1471-2458-9-67 19236726 PMC2656537

[B24] The Millennium Development Goals Report United Nations 2008 http://mdgs.un.org/unsd/mdg/Resources/Static/Products/Progress2008/MDG_Report_2008_En.pdf

