# Supplementary material for: Spatio-temporal analysis of mortality among children under the age of five in Manhiça (Mozambique) during the period 1997-2005
Source: Int J Health Geogr. 2011 Feb 18;10:14. doi: 10.1186/1476-072X-10-14 (PMC3050678; doi:10.1186/1476-072X-10-14)
Supplement: Additional file 1 — SAS syntax details. SAS syntax details for the Poisson regression mixed models parameter estimates. [file 1476-072X-10-14-S1.DOC]

# SAS syntax details

Generalized Linear Mixed Models can be estimated in SAS (v9.1) via the GLIMMIX procedure. However the improper conditional autoregressive model (ICAR) to structure the covariance matrix of random effects as defined in Additional file 1 is not an available option in GLIMMIX. Nevertheless we can reformulate the ICAR model as $\mathbf{b}_{j}=\mathbf{Zb}_{j}^{*}$, with $\mathbf{Z}=\left(\mathbf{Q}^{-}\right)^{\mathbf{1/2}}$ and $\mathbf{b}_{j}^{*}\text{\textasciitilde MVN}\left(\mathbf{0},\sigma_{S}^{2}\mathbf{I}\right)$ and call a GLIMMIX procedure as follows:

**proc** **glimmix** data=st.mortality initglm maxopt=**50** IC=PQ;

class neighbourhood q_num_constructions;

model death = year year_2 q_num_constructions/ dist=poisson offset=ln_expected solution ddfm=sat;

random Z1-Z115 / subject=neighbourhood type=toep(**1**) s;

random _residual_;

**run**;

where Z1-Z115 are the columns of the Z matrix. A toeplitz structure with order equal to 1 for the random effects covariance matrix is applied to specify the same variance component for all the random effects. The random _residual_ statement is included to estimate the dispersion parameter.

If in addition exchangeable random effects are included to account for neighbourhood-specific time trend curvatures the GLIMMIX procedure is written as follows:

**proc** **glimmix** data=st.mortality initglm maxopt=**50** IC=PQ;

class neighbourhood q_num_constructions;

model death = year year_2 q_num_constructions/ dist=poisson offset=ln_expected solution ddfm=sat;

random Z1-Z115 / subject=neighbourhood type=toep(**1**);

random year year_2 /sub=neighbourhood;

random _residual_;

**run**;
